# Aortic endograft infection by *Mycobacterium abscessus* subsp. *massiliense* with acquired clarithromycin resistance: a case report

**DOI:** 10.1186/s12879-023-08702-1

**Published:** 2023-10-17

**Authors:** Yutaro Akiyama, Noriko Iwamoto, Keisuke Kamada, Atsushi Yoshida, Asami Osugi, Satoshi Mitarai, Tetsuya Suzuki, Kei Yamamoto, Maki Nagashima, Tetsuya Horai, Norio Ohmagari

**Affiliations:** 1 Disease Control and Prevention Center, National Center for Global Health and Medicine, 1-21-1, Toyama, Shinjuku-ku, Tokyo, 162-8655 Japan; 2 Department of Mycobacterium Reference and Research, The Research Institute of Tuberculosis, Japan Anti-Tuberculosis Association, 3-1-24 Matsuyama, Kiyose-shi, Tokyo, 204-8533 Japan; 3 Department of Infectious Diseases, Tokyo Metropolitan Geriatric Hospital and Institute of Gerontology, 35-2 Sakae-cho, Itabashi-ku, Tokyo, 173-0015 Japan; 4 Department of Cardiovascular Surgery, National Center for Global Health and Medicine, 1-21-1, Toyama, Shinjuku-ku, Tokyo, 162-8655 Japan

**Keywords:** *Mycobacterium abscessus* subsp. *massiliense*, Aortic endograft infection, Case report, Macrolide resistance

## Abstract

**Background:**

*Mycobacterium abscessus* subsp. *massiliense* (MMA) comprises a group of non-tuberculous, rapidly growing mycobacteria. Although MMA can cause pulmonary diseases, surgical site infections, and disseminated diseases, aortic endograft infection has not been reported. Here, we describe the first case of aortic endograft infection caused by MMA.

**Case presentation:**

Two months after stent-graft insertion for an abdominal aortic aneurysm, an 85-year-old man was admitted with fever and abdominal pain and was diagnosed with aortic endograft infection. Despite 14 days of meropenem and vancomycin intravenous administration, periaortic fluid pooling increased as compared to that before antibiotic administration. The abscess was drained, and fluorescent acid-fast staining of the abscess fluid revealed bacilli. We conducted genetic tests on the genes *hsp65*, *rpoB*, and *sodA*, performed Whole Genome Sequencing (WGS), and identified the organism as MMA. Intravenous imipenem–cilastatin (IPM/CS), amikacin (AMK), and oral clarithromycin (CAM) were administered. After 2 months, oral CAM and sitafloxacin were administered because the abscess had decreased in size. However, after 6 weeks, the abscess increased in size again. Antimicrobial susceptibility testing of the drainage fluid from the abscess resulted in the isolation of an MMA strain that had acquired resistance to CAM. Intravenous IPM/CS, AMK, and oral linezolid were added to the treatment regimen along with oral CAM and STFX. However, he was not fully cured and died 6 months later. Neither the full-length erythromycin ribosome methyltransferase (*erm*)(41) gene nor the *rrl* or *rpIV* gene mutations were found by Sanger sequencing in the pre- and post-treatment strains. Whole-genome sequence analysis of the post-treatment strain revealed mutations in genes with no previous reports of association with macrolide resistance.

**Conclusions:**

Aortic endograft infection caused by MMA strain is extremely rare; nonetheless, MMA should be suspected as the causative microorganism when broad-spectrum antimicrobials are ineffective.

**Supplementary Information:**

The online version contains supplementary material available at 10.1186/s12879-023-08702-1.

## Background

*Mycobacterium abscessus* (*M*. *abscessus*) comprises a group of non-tuberculous, rapidly growing mycobacteria (RGM) and consist of three subspecies: *M*. *abscessus* subsp. *abscessus*, *M*. *abscessus* subsp. *bolletii,* and *Mycobacterium abscessus* subsp. *massiliense* (MMA) [1]*. Mycobacterium abscessus* subsp. *abscessus* and *bolletii* exhibit macrolide resistance with a full-length functional erythromycin ribosome methyltransferase (*erm*)(41), whereas MMA exhibits macrolide susceptibility with a truncated, nonfunctional *erm*(41) [2]. *Mycobacterium abscessus* subsp. *massiliense* can be differentiated from the other two subspecies by a polymerase chain reaction (PCR) analysis of *erm*(41) as MMA has a shorter *erm*(41). The *erm*(41) DNA amplified from MMA are 397-bp long, whereas the *erm*(41) DNA amplified from the other 2 subspecies are 673-bp long [2]. The *rrl* mutation is associated with acquired resistance to macrolides in RGM [3]. Other mutations associated with macrolide resistance have also been reported in MMA, including mutations in the *rpIV* gene [4].

Although MMA can cause pulmonary diseases, surgical site infections (SSI), and disseminated diseases [1], no cases of aortic endograft infection have been reported. Here, we describe the first case of aortic endograft infection caused by MMA. During the clinical course, the bacteria acquired macrolide resistance, and mutations were observed in genes with no previous reports of associations with macrolide resistance.

## Case presentation

**Fig. 1:**
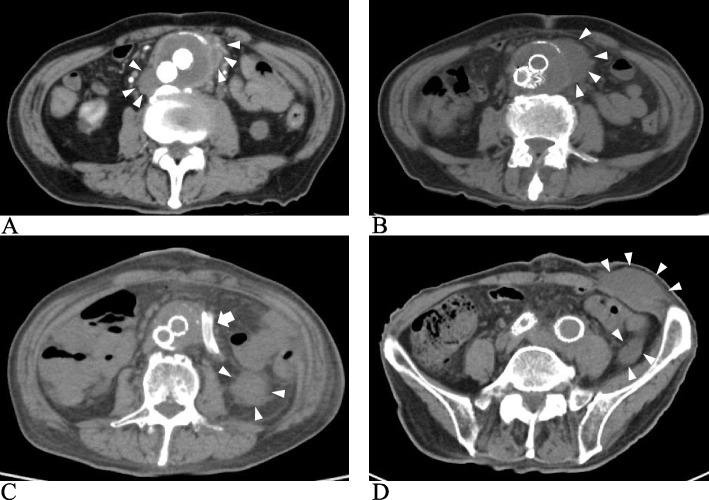
The results of computed tomography scan. **A** Before initiating antimicrobial therapy. **B** After 14 weeks of antibiotic therapy, periaortic fluid pooling became larger than that before antibiotics administration. **C**-**D** Three days after drainage surgery, the abscess expanded and spread along the drainage tube and formed a subcutaneous abscess around the drain insertion site. White arrowheads represent the abscess and white arrows represent the drainage tube

An 85-year-old man was hospitalized with a 4-day history of fever and abdominal pain. He had undergone stent-graft insertion for an abdominal aortic aneurysm 2 months prior. Computed tomography (CT) revealed periaortic fluid collection consistent with stent-graft placement (Fig. 1A). Based on the clinical information, he was diagnosed with aortic endograft infection and administered infusion of meropenem (1 g twice daily) and vancomycin (0.4 g twice daily).

**Fig. 2:**
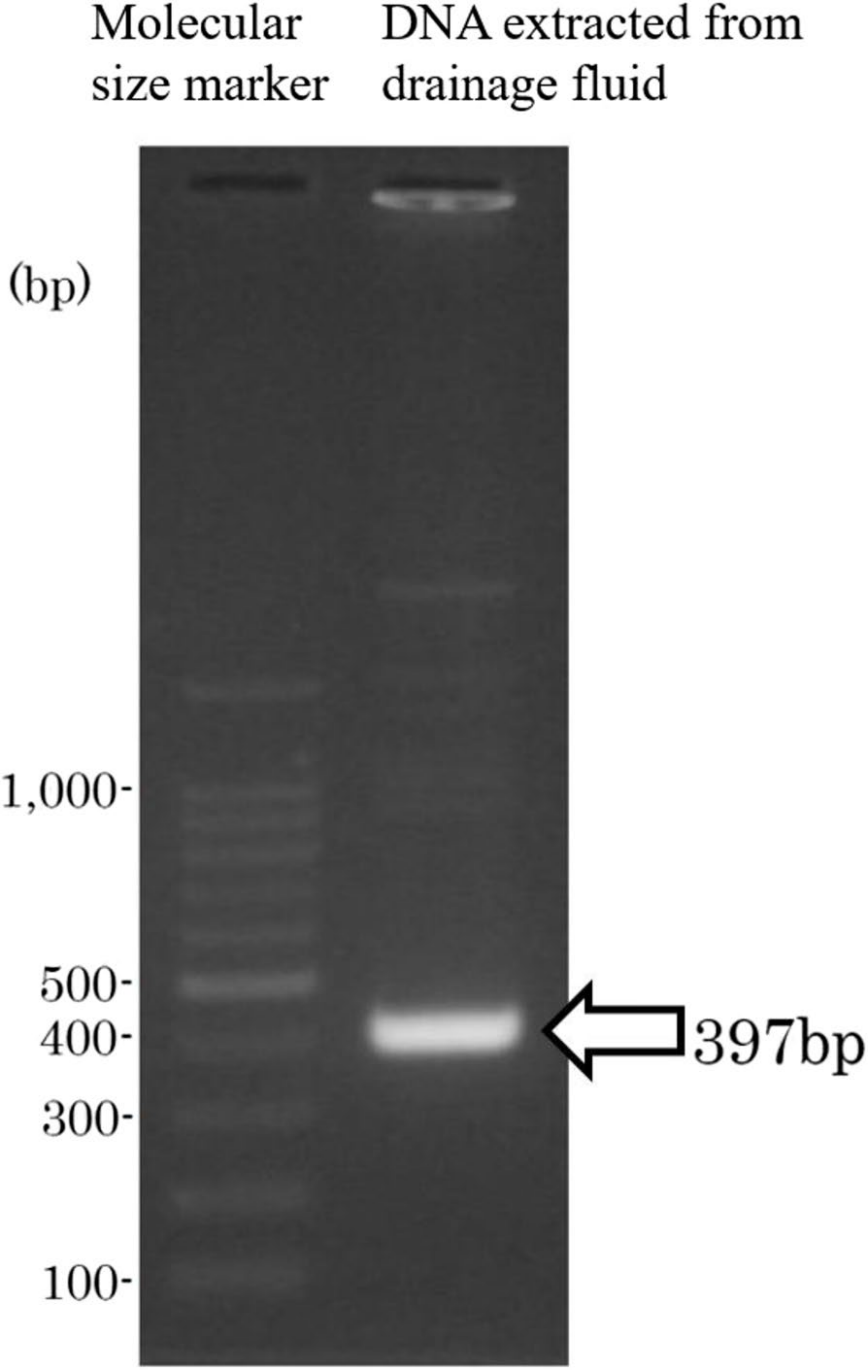
Agarose gel electrophoresis of DNA extracted from drainage fluid. The white arrow shows amplification of the 397 bp-long erythromycin ribosome methyltransferase gene. Full-length gels are presented in Supplementary Figure 1

Despite 14 weeks of antibiotic therapy, periaortic fluid accumulation increased as compared to that before antibiotic administration (Fig. 1B). Surgical abdominal drainage was performed, and an indwelling drain was inserted. Bacterial culture of the drainage fluid gave negative results on sheep blood agar, BTB lactose agar, and chocolate agar at 35 ℃ in 5% CO2 for 24–48 hours. Three days after the drainage surgery, the periaortic fluid increased again and spread along the drainage tube, forming a subcutaneous abscess around the drain insertion site (Fig. 1C, D). The abscess was drained, and fluorescent acid-fast staining of the abscess fluid revealed bacilli. The organism was identified as *Mycobacterium abscessus* by nucleic acid sequencing of housekeeping genes (16S rRNA, *rpoB*, *hsp65*, *sodA*) (Table 1). The PCR product length of *erm*(41) was only 397 bp, which indicates deletion of this gene, according to a previous study [2] (Fig. 2). Thus, the organism was identified as MMA. The same organism was isolated from the periaortic fluid specimens. In addition, the first drainage sample was positive on 7-day culture in thioglycolate broth, and the organism was identified as MMA. Based on these results, the patient was diagnosed with aortic endograft infection with MMA.

**Table 1:** Analysis results of 16S rRNA, *rpoB*, and *hsp65* sequence

**RANK**	**Name**	**Strain**	**Accession**	**Pairwise Similarity(%)**	**Mismatch/Total nt**
1. 16S rRNA (sequence similarity analysis with EZ Bio Cloud): *Mycobacterium* species					
1	*Mycobacterium abscessus* subsp. *bolletii*	BD	AHAS01000006	99.93	1/1438
2	*Mycobacterium abscessus* subsp. *massiliense*	CCUG 48898	AKVF01000003	99.93	1/1438
3	*Mycobacterium abscessus* subsp. *abscessus*	ATCC 19977	CU458896	99.93	1/1438
4	*Mycobacterium chelonae* subsp. *bovis*	QIA-37	CP010071	99.86	2/1438
5	*Mycobacterium saopaulense*	EPM 10906	CP010271	99.86	2/1438
6	*Mycobacterium chelonae* subsp. *gwanakae*	MOTT36W	CP031516	99.86	2/1438
7	*Mycobacterium franklinii*	CV002	MAFQ01000001	99.65	5/1438
2. *rpoB* (sequence similarity analysis with BLAST): *Mycobacterium abscessus* species					
*Mycobacterium abscessus* subsp. *massiliense*		CCUG 48898	AP014547	99.72	724/726
*Mycobacterium abscessus* subsp. *bolletii*		CIP 108541	AY859692	98.21	713/726
3. *hsp65* (sequence similarity analysis with BLAST): *Mycobacterium abscessus* species					
*Mycobacterium abscessus* subsp. *massiliense*		CCUG 48898	AP014547	100	421/421
*Mycobacterium abscessus* subsp. *bolletii*		CIP 108541	AY859675	99.29	418/421

**Fig. 3:**
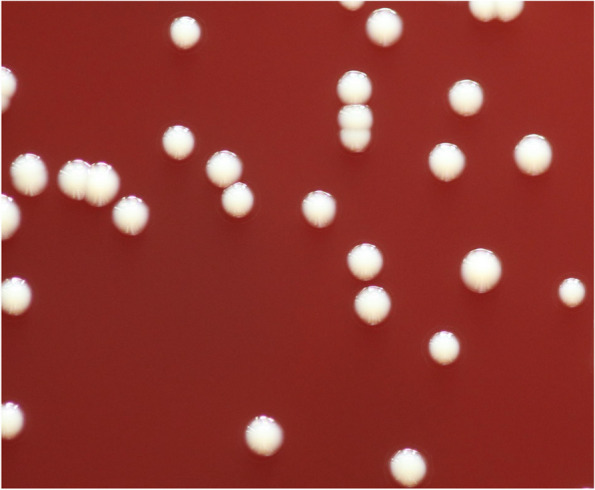
Colonies of post-treatment strains. Post-treatment strains cultured on sheep blood agar

The antibiotic regimen was changed to intravenous imipenem-cilastatin (IPM/CS) 500 mg four times a day, amikacin (AMK) 300 mg every other day (Ccr 26.3ml/min), and oral clarithromycin (CAM) 400 mg twice daily. Two months after this treatment, only oral CAM 400 mg twice daily and sitafloxacin (STFX) 100 mg once daily were administered because CT showed a decreased in the size of the abscess. Although the MIC of STFX is lower than that of other fluoroquinolones, the breakpoint of STFX is not well-defined. (Table 2). However, after 6 weeks of treatment, the abscess around the aortic aneurysm increased in size again, and a new abscess appeared around the bilateral iliac arteries and veins. Antimicrobial susceptibility testing of the drainage fluid from the abscess resulted in the isolation of an MMA strain that had acquired resistance to CAM (Table 2). Intravenous IPM/CS 500 mg four times a day, AMK 300 mg daily, and oral linezolid (LZD) 600 mg twice daily were added to the treatment regimen along with oral CAM and STFX. LZD was discontinued within 1 week because of thrombocytopenia. The four-antibiotic combination therapy and abscess drainage were continued for 4 months, but the size of the abscesses did not change. On day 286 of hospitalization, he was discharged to a nursing home with a drainage tube, and the treatment with oral CAM, STFX, and intravenous AMK was continued by a visiting physician. Six months after discharge from the hospital, the patient died of an unknown cause. Neither the full-length *erm*(41) gene nor the *rrl* gene mutation was found by Sanger sequencing in the pre- and post-treatment strains. The reserved pre- and post-treatment strains were cultured on sheep blood agar (Fig. 3) for bacterial recovery and DNA extraction, and whole-genome sequence analysis (WGS) was performed on a NextSeq550 sequencer (Illumina, San Diego, CA). WGS of the post-treatment strains revealed two indels and one single nucleotide polymorphism (SNP) (Table 3). There was one SNP between the pre- and post-treatment strains, and the average nucleotide identity was 99.99%; thus, both the strains were considered identical. Reinfection with another species of MMA was ruled out based on the WGS results.

**Table 2:** Susceptibility test results of *Mycobacterium abscessus* subspecies *massiliense*

	**Strain A prior to treatment initiation**		**Strain B after 2 months of treatment**	
Antimicrobial agent	MIC (μg/mL)	Susceptibility	MIC (μg/mL)	Susceptibility
Linezolid	16	I	16	I
Clarithromycin (ERT)	0.25	S	**1**	S
Clarithromycin (LRT)	0.5	S	**>32**	R
Amikacin	8	S	8	S
Tobramycin	16	R	8	R
Imipenem	16	I	4	S
Ciprofloxacin	16	R	16	R
Sitafloxacin	2	-	1	-
Moxifloxacin	>8	R	8	R
Cefoxitin	32	I	16	S
Minocycline	32	R	32	R
Sulfamethoxazole/ trimethoprim	152/8	R	152/8	R

*S* Susceptible, *I* Intermediate, *R* Resistant

*MIC* Minimum inhibitory concentration

*ERT* Early reading time (3–5 days), *LRT* Late reading time (14 days)

**Table 3:** Result of whole genome sequence analysis of two strains of *Mycobacterium abscessus* subspecies *massiliense*

Position	Gene	variant	Pre-treatment	Post-treatment	Codon_pre-treatment	Codon_post-treatment
725354	aminodeoxychorismate lyase	frame_shift	TG	T	-	-
2181851	LLM class flavin-dependent oxidoreductase	missense	T	C	CTG	CCG
3515411	Not Applicable	intergenic	C	CG	-	-

*LLM* Luciferase-like monooxygenase

## Discussion

This case is noteworthy because it presents three instructive points. First, aortic graft infection caused by MMA is extremely rare. Second, the identification of MMA from an aortic graft infection treated with prolonged antimicrobial therapy is clinically meaningful in terms of selection of antimicrobials. Third, the MMA strain acquired resistance to clarithromycin during the treatment course.

Aortic endograft infection is rare and has a poor prognosis, with an incidence rate of 0.43–2.0% [5–10] and a mortality rate of 11–50% [5, 6, 8–12]. The causative pathogens include *Staphylococcus* sp., *Streptococcus* spp., *Propionibacterium* spp., *Escherichia coli*, and *Enterobacter cloacae*, which are culture-negative in 20–55% of cases [5–7, 9–11]. Aortic endograft infection with nontuberculous mycobacteria (NTM) is extremely rare. Mizoguchi et al. [13] reported an endovascular device infection with an iliopsoas abscess caused by *Mycobacterium tuberculosis* var. *bovis* after intravesical bacillus Calmette**–**Guérin therapy; Shaikh et al. [14] reported an aortic graft infection with a *Mycobacterium avium* complex in a patient with a human immunodeficiency virus infection; and Plate et al. [15] reported an aortic endograft infection with *Mycobacterium chimaera* associated with an outbreak in the high-care units. To the best of our knowledge, this is the first report of aortic endograft infection with MMA. This case report highlights that despite the rarity, clinicians should still consider the possibility of mycobacterial infection, in cases of no improvement following treatment with broad-spectrum antibiotics.

In this case, the route of infection was unknown. Patients at risk for NTM infections are immunocompromised patients or those with pre-existing pulmonary conditions [16]. However, our patient was not immunocompromised and had no pulmonary abnormalities. Nontuberculous mycobacteria can be a causative agent of SSI, and outbreaks due to the contamination of medical devices have been reported. For example, ultrasound gel contaminated by MMA on plastic trays, [17] and *Mycobacterium wolinskyi* [18] and *Mycobacterium chimaera* [19, 20] transmission through heater-cooler-devices for extracorporeal circulation. Six months after this case, another patient who underwent ascending aorta replacement at our hospital, developed bacteremia due to MMA*.* We conducted microbial surveillance in the operating room environment, which included carts, shelfs, personal computers, ultrasonic probes, tube holders, and the hands of doctors and nurses. However, MMA was not detected anywhere, and no common devices were used between the two cases (extracorporeal circulation was not used in our case).

The MMA strain isolated in this case had acquired resistance to clarithromycin 2 months after the initiation of clarithromycin treatment. Two gene mutations related to macrolide susceptibility in RGM have been reported: *erm*(41) is involved in macrolide-induced resistance, and *rrl* is associated with acquired resistance to macrolides [3]. According to the WGS of the pre- and post-treatment strains, neither reinfection with another species of MMA with full-length *erm*(41) nor acquisition of the *rrl* mutation from the same species was found. Other mutations associated with macrolide resistance in MMA have been also reported, including mutations in the *rpIV* gene [4]. WGS of the post-treatment strains revealed a frameshift mutation in the gene encoding aminodeoxychorismate lyase and a missense mutation (CTG→CCG) in the gene encoding the luciferase-like monooxygenase class flavin-dependent oxidoreductase, which was not associated with *erm*(41), *rrl,* and *rpIV* mutations. Aminodeoxychorismate lyase catalyses the following chemical reaction, “4-amino-4-deoxychorismate = 4-aminobenzoate + pyruvate” [21]. Luciferase-like monooxygenase class flavin-dependent oxidoreductase transfers one oxygen atom of an oxygen molecule to a substrate while reducing the other oxygen atom to water [22]. The effect of these enzymatic changes on MMA is not known.

There were three mutation sites between the two strains shown by WGS, but these were in regions not associated with known macrolide susceptibility. It should be clearly stated that the cause of macrolide resistance in this instance remains uncertain. Inferring the mechanism of macrolide resistance solely from the function of the mutated genes in this case is challenging. Moreover, it cannot be definitively ruled out that these mutations may not be involved in drug resistance at all. In this case, the mechanism by which the MMA strain acquired macrolide resistance remains unknown. However, new gene mutations leading to clarithromycin resistance in MMA have been reported [23, 24], and further WGS results may clarify the association between the genetic variants in our case and clarithromycin resistance.

## Conclusion

We report a case of infected aortic aneurysm caused by MMA. Aortic endograft infection caused by an MMA strain is extremely rare, but MMA should be suspected as the causative microorganism when broad-spectrum antimicrobials are ineffective. During the clinical course, the bacteria acquired macrolide resistance and mutations were observed in genes with no report of associations with macrolide resistance.

### Supplementary Information

**Additional file 1: Supplementary Figure 1.** 

## Data Availability

The datasets used and/or analysed during the current study available from the corresponding author on reasonable request.

## Abbreviations

RGM: Rapid growing mycobacteria
MMA: *Mycobacterium abscessus* subspecies *massiliense*
*erm*: Erythromycin ribosome methyltransferase
SSI: Surgical site infection
CT: Computed tomography
IPM/CS: Imipenem-cilastatin
AMK: Amikacin
CAM: Clarithromycin
STFX: Sitafloxacin
LZD: Linezolid
WGS: Whole genome sequence analysis
SNP: Single nucleotide polymorphism
NTM: Non-tuberculous mycobacteria
