# Supplementary figures and images for: Aortic endograft infection by Mycobacterium abscessus subsp. massiliense with acquired clarithromycin resistance: a case report
Source: BMC Infect Dis. 2023 Oct 17;23:694. doi: 10.1186/s12879-023-08702-1 (PMC10583484; doi:10.1186/s12879-023-08702-1)

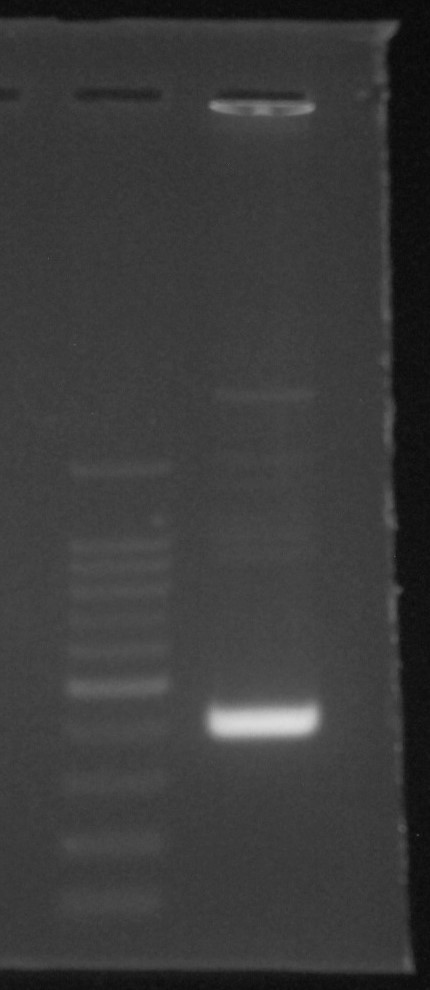

Supplement: Supplementary file 1 — Additional file 1: Supplementary Figure 1. [file 12879_2023_8702_MOESM1_ESM.png]
